# The Current State of Digital Variance Angiography: A Literature Review

**DOI:** 10.7759/cureus.116912

**Published:** 2026-09-25

**Authors:** Sierra Scott, Jonathan Platosz, Shannon N Smith, Sydney Elness, Suzanne I Riskin

**Affiliations:** 1 Department of Foundational Sciences, Nova Southeastern University Dr. Kiran C. Patel College of Osteopathic Medicine, Clearwater, USA

**Keywords:** angiography, contrast to noise ratio, digital subtraction angiography, digital variance angiography, endovascular surgery, interventional radiology, kinetic imaging, radiation dose reduction, signal to noise ratio, vascular imaging

## Abstract

Digital variance angiography (DVA), also referred to as kinetic imaging angiography, is an imaging technique that enhances vascular visualization by analyzing contrast flow motion. Although digital subtraction angiography (DSA) remains the current standard of care, DVA has demonstrated potential improvements in image quality and reductions in both radiation exposure and contrast use. This literature review aimed to evaluate currently available literature focusing on the use of DVA during angiographic procedures. The review was conducted using PubMed, EMBASE, and Web of Science, with the process being reflected in a Preferred Reporting Items for Systematic Reviews and Meta-Analysis (PRISMA) flowchart. Across multiple clinical applications, DVA consistently demonstrated higher objective image quality metrics including contrast-to-noise ratio (CNR) and signal-to-noise ratio (SNR) as compared with DSA. Several studies reported that expert reviewers observed improved subjective image quality and substantial reductions in radiation exposure. In addition, DVA has also been shown to facilitate reduced use of contrast, potentially decreasing contrast-related complications and healthcare costs. Collectively, current evidence suggests that DVA represents a promising advancement in angiographic imaging, with the potential to deliver superior image quality with reduced radiation exposure and contrast use.

## Introduction and background

Angiography aims to evaluate blood vessels by injecting iodinated contrast agents under fluoroscopy [1]. With real-time visualization, fluoroscopy enables operators to assess vascular anatomy and abnormalities during both diagnostic and interventional procedures [1]. Such abilities are essential for making intra-procedural clinical decisions. This approach is widely used for disease management in both outpatient and inpatient settings. Interventional cardiology uses angiography for coronary artery disease, interventional neurology for stroke, and vascular and interventional radiology for embolization, angioplasty, and stent placement [1].

Digital subtraction angiography (DSA) subtracts pre-contrast images from contrast-enhanced images, removing background structures such as bone and soft tissue [2]. This technique is widely used and improves the visualization of contrast-filled vessels [2]. DSA is sensitive to patient movement between image acquisitions, which can introduce motion artifacts that degrade image quality [2]. Postprocessing techniques such as image registration can reduce these effects, although it does not fully eliminate them.

Digital variance angiography (DVA), also known as kinetic imaging angiography, is a novel technique for imaging the vasculature. Unlike DSA, DVA calculates the standard deviation in X-ray attenuation at each collected pixel [3]. This approach uses more information from raw, unsubtracted image sequences than traditional subtraction techniques. By emphasizing changes in signal from moving contrast and minimizing background noise, DVA can enhance image quality. It represents a potential alternative to conventional subtraction-based angiographic methods [3].

## Review

Methods

This literature review aimed to evaluate currently available literature focusing on the use of DVA during angiographic procedures, focusing on clinical, dosimetric, and economic outcomes. PubMed, EMBASE, and Web of Science were searched for articles addressing kinetic imaging techniques in angiography, using the keywords with Boolean modifiers, ‘kinetic imaging,’ ’kinetic imaging angiography,’ and ’digital variance angiography’ ensuring the inclusion of articles with at least one of the specified terms. Inclusion criteria were articles on the topic using keywords, articles published between January 1, 2019, and December 31, 2025, and articles published in English. Abstracts were excluded from inclusion in the literature review. Table 1 details the search strategy utilized for this review.

**Table 1:** Search strategies used in the retrieval of articles for review QALY = quality-adjusted life-year, RR = relative risk.

Database	Search Terms	Articles Yielded
PubMed	("kinetic imaging" tiab OR "kinetic imaging angiography" tiab OR "digital variance angiography" tiab) AND englishla AND ("2019/01/01"pdat : "2025/12/31"pdat)	42
EMBASE	('kinetic imaging':ti,ab OR 'kinetic imaging angiography':ti,ab OR 'digital variance angiography':ti,ab) AND english/lim AND 2019-2025/py	32
Web of Science	TS=("kinetic imaging" OR "kinetic imaging angiography" OR "digital variance angiography") AND LA=(English) AND PY= (2019-2025)	37

The initial search yielded a total of 111 publications. After Tier 1 screening of titles, and abstracts, articles were then screened by full texts for relevance. A total of 68 articles were excluded for duplication, 26 for lack of relevance to human vasculature imaging, and one for wrong study design. Ultimately, 16 articles were included in this review. All 16 articles that met established inclusion and exclusion criteria were then assessed for bias using the Joanna Briggs Institute (JBI) Critical Appraisal Tools. Following detailed discussions and utilization of appropriate checklists from the JBI, all 16 articles were deemed to have low or moderate risk of bias and were therefore included in the review. The results of the critical appraisal utilizing the JBI tools and discussed concerns for each article can be found in Table 2.

**Table 2:** Results from the assessment of bias using JBI appraisal tools JBI = Joanna Briggs Institute.

Author (Year)	JBI Critical Appraisal Tool	Appraisal Concerns
Nyárády et al., 2025 [3]	JBI analytical cross-sectional studies	Selection bias, small sample size
Lucatelli et al., 2023 [4]	JBI analytical cross-sectional studies	Selection bias, small sample size
Bérczi et al., 2026 [5]	JBI analytical cross-sectional studies	Small sample size
Gyánó et al., 2021 [6]	JBI quasi-experimental studies	Small sample size, non-randomized study
Góg et al., 2024 [7]	JBI analytical cross-sectional studies	Multiple measurements taken from same patient causing possible artificial inflation of sample size
Alizadeh et al., 2023 [8]	JBI analytical cross-sectional studies	Small sample size, multiple measurements taken from same patient causing possible artificial inflation of sample size
Gyánó et al., 2020 [9]	JBI case series	Selection bias
Óriás et al., 2019 [10]	JBI quasi-experimental studies	Small sample size
Gyánó et al., 2019 [11]	JBI quasi-experimental studies	Multiple measurements taken from same patient causing possible artificial inflation of sample size
Bastian et al., 2021 [12]	JBI case series	Small sample size
Sótonyi et al., 2023 [13]	JBI randomized controlled trials	Lowest evidence of bias
Elek et al., 2024 [14]	JBI economic evaluations	Indirect evidence
Schürmann et al., 2025 [15]	JBI cohort studies	Selection bias
Thomas et al., 2022 [16]	JBI analytical cross-sectional studies	Non-randomized
Óriás et al., 2020 [17]	JBI quasi-experimental studies	Small sample size
Ansaripour et al., 2024 [18]	JBI economic evaluations	Indirect evidence

Figure 1 presents the screening process utilized for this review in a Preferred Reporting Items for Systematic Reviews and Meta (PRISMA) flowchart. 

**Figure 1:**
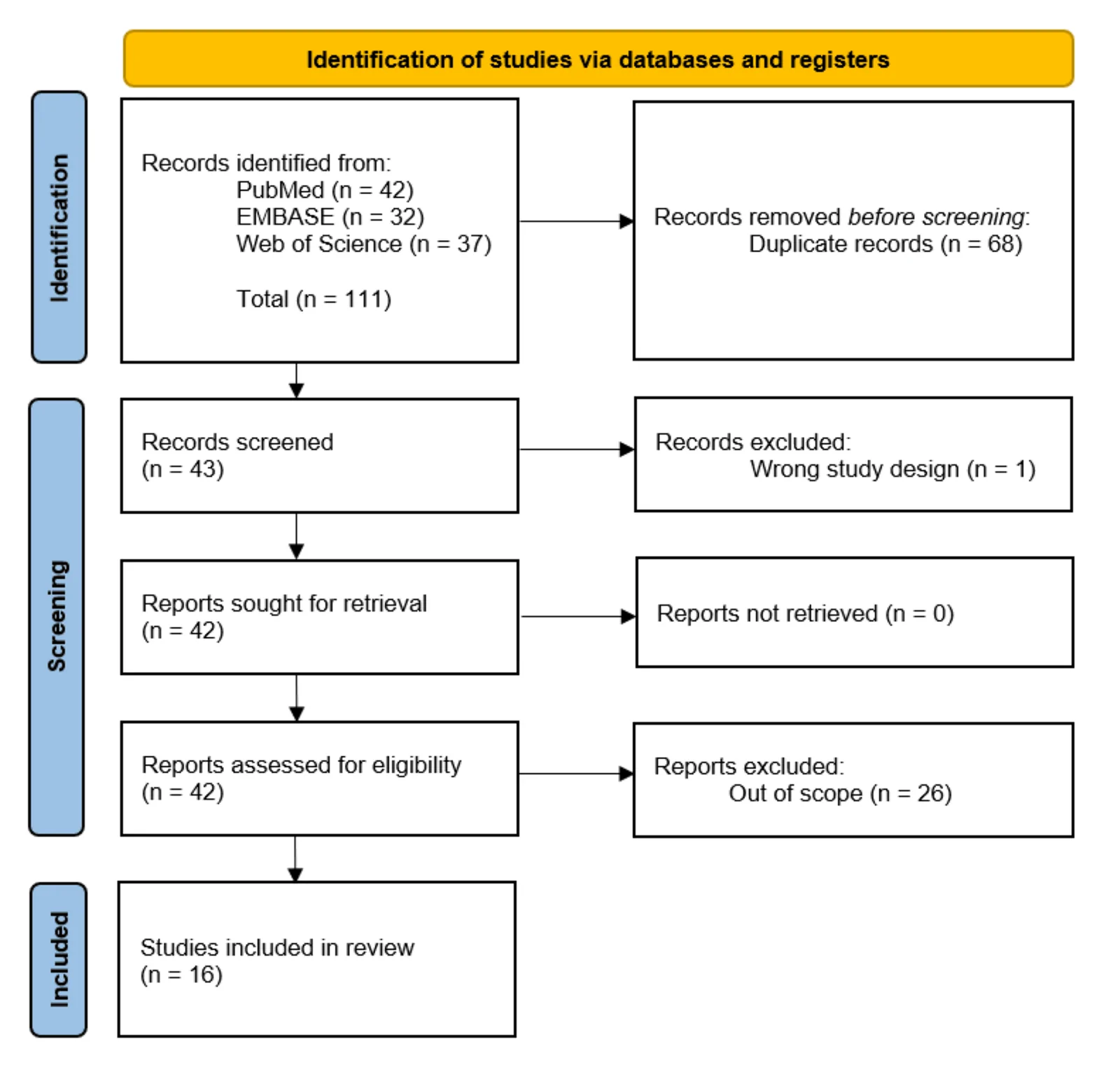
Preferred Reporting Items for Systematic Reviews and Meta-Analyses (PRISMA) diagram of the search strategy to determine studies utilized.

Results

The results of the articles included in this review are depicted below in Table 3.

**Table 3:** Summary table of the 16 included articles in this review. AKI = acute kidney injury, AVM = arteriovenous malformation, BPH = benign prostatic hyperplasia, ccDSA = color-coded digital subtraction angiography, ccDVA = color-coded digital variance angiography, CLTI = critical limb-threatening ischemia, CNR = contrast-to-noise ratio, DAP = median total dose area product, DICOM = Digital Imaging and Communications in Medicine, DSA = digital subtraction angiography, DVA = digital variance angiography, ICM = iodinated contrast medium, LAR = lifetime attributable risk, LD = low-dose, maxDSA = maximal intensity projection of DSA, n/a = not applicable, ND = normal-dose, PAD = peripheral artery disease, PAE = prostate artery embolization, sumDSA = standard mask subtraction method, TACE = transarterial chemoembolization, TTP = time-to-peak, UFE = uterine fibroid embolization.

Author, Year	Sample Size	Intervention	Study Design	Relevant Conclusions
Nyárády et al., 2025 [3]	10	Endovascular intervention for AVMs	Retrospective observational study; DVA was compared with DSA using CNR, and five experts rated image quality on a four-point Likert scale.	DVA had a significantly higher CNR as compared with DSA, but was not superior in clinician visual assessment.
Lucatelli et al., 2023 [4]	25	Liver TACE	Retrospective observational study; five experts compared DSA and DVA, rating image quality on a four-point Likert scale.	DVA demonstrated higher image quality as compared to DSA and had fewer instances of non-visible lesions and feeding arteries. 80% of readers preferred DVA over DSA.
Bérczi et al., 2026 [5]	56	UFE	Retrospective observational study; DVA was compared with DSA using CNR, and three experts rated image quality on a four-point Likert scale.	DVA images showed a two-fold higher median CNR as compared with DSA, which was significant. Subjective visual comparison showed no significant clinical difference between DVA and DSA for UFE.
Gyánó et al., 2021 [6]	30	Angiography for PAD	Prospective observational study; images were obtained using ND and LD contrast protocols for both DVA and DSA for CNR calculation, and seven experts rated image quality on a five-point Likert scale.	DVA images when statistically analyzed against their same-dose DSA counterpart received higher visual evaluation scores in all anatomical regions, except for the ND femoral group which had no significant difference. LD-DVA images, when statistically compared to ND-DSA, had significantly higher CNR ratios in all regions.
Góg et al., 2024 [7]	19	Endovascular intervention for CLTI	Retrospective observational study; comparison of ccDSA and ccDVA using time-related parameters, TTP and change in passage time.	Both time-related parameters in ccDVA were essentially the same as ccDSA.
Alizadeh et al., 2023 [8]	30	PAE for BPH	Retrospective observational study; DSA was compared with DVA and ccDVA images using CNR, and four experts rated image quality on a four-point Likert scale.	DVA images showed a more than four-fold higher CNR value than DSA, which was significant. DVA images had a significantly higher visual evaluation score than DSA. Evaluation of paired DSA and ccDVA images yielded a significant preference for ccDVA images with the best outcome for tissue blush appearance.
Gyánó et al., 2020 [9]	4	Lower-limb CO_2_ angiography for PAD	Case series; five experts compared DSA and DVA, rating image quality on a five-point Likert scale.	84.8% of single DVA images were rated higher in image quality, while DSA only had a higher score in 8.7% of single images. When compared directly, DVA achieved a score of 85.2% for diagnostic value and 83.9% for usefulness for therapeutic decision making, which was significant.
Óriás et al., 2019 [10]	24	Lower-limb CO_2_ angiography	Prospective observational study; DVA was compared with DSA using SNR, and four experts rated image quality on a four-point Likert scale. Two SNRs were calculated: SNR of DVA to sumDSA and SNR of DVA to maxDSA.	SNR was consistently higher in favor of DVA regardless of region, clinical center, or ratio type. DVA yielded a significantly higher image quality score than DSA, regardless of anatomic location.
Gyánó et al., 2019 [11]	42	Lower-limb angiography for PAD	Prospective observational crossover study; four image types were generated and compared (raw DSA, postprocessed DSA, raw kinetic, kinetic with postprocessing) through calculating SNR and by six experts evaluating paired images based on image detail, visibility of anatomical structures, and diagnostic usefulness.	When comparing SNR values, postprocessed DSA showed improvement over raw DSA; however, raw kinetic imaging still demonstrated a 2.3-fold higher SNR across all regions compared to postprocessed DSA and a 3.3-fold increase compared to raw DSA. In overall image comparison, postprocessed kinetic imaging was rated superior in 69% of cases, with statistically significant improvements noted in the talocrural and popliteal regions.
Bastian et al., 2021 [12]	12	Lower extremity angiography for PAD in patients with metal implants	Retrospective observational study; DVA was compared with DSA using CNR, and five experts rated image quality through a five-point Likert scale and two questionnaires.	When image pairs near implants were compared, experts preferred DVA images 83.75% of the time with 79.6% agreement. DVA images received significantly higher Likert scores compared to DSA images.
Sótonyi et al., 2023 [13]	114	Lower-limb angiography for PAD	Prospective randomized controlled trial; patients were randomly assigned to LD and ND groups. The ND group's images were processed using DSA, whereas the LD group's images were processed using DSA and two different DVA algorithms (DVA1 and DVA2). Six experts rated image quality on a five-point Likert scale.	The DAP and DSA-related DAP reductions in the LD group, when compared to the ND group, were statistically significant. LD-DSA images received significantly lower scores from readers compared to ND-DSA images. LD-DVA1 images received similar scores to ND-DSA images, and LD-DVA2 images received significantly higher scores than ND-DSA images in all regions except the aortoiliac region, with a significant difference only in the popliteal region.
Elek et al., 2024 [14]	107	Lower-limb angiography for PAD	Retrospective observational study; dosimetric exposure data from another study’s DICOM data [13] was extrapolated, and a Monte Carlo simulation was performed to calculate effective dose and LAR in patients exposed to DSA and DVA interventions.	The collective effective dose was reduced by 58% in the DVA group compared to the DSA group. LAR of cancer incidence was reduced by 25%-75% and LAR of cancer mortality was reduced by 51%-84% in favor of DVA.
Schürmann et al., 2025 [15]	370	General endovascular interventions	Retrospective observational study; data from the institutions’ dose management system was used to calculate DAP and CNR for each DSA acquisition in patients who were exposed to LD and ND protocol. DVA images were generated from the existing raw data and organized by exposure to LD and ND protocol.	A significant decrease in the median DAP for LD-DSA acquisitions was demonstrated by utilizing LD-DVA across all regions. DVA had superior CNR values when compared with ND- and LD-DSA groups.
Thomas et al., 2022 [16]	28	Endovascular intervention for PAD	Prospective observational study; DSA was compared to two DVA algorithms (DVA1 and DVA2) using CNR, and six experts rated image quality on a five-point Likert scale. As an additional variable, participants received either ICM or CO_2_.	DVA images produced significantly higher CNR values as compared with DSA. For the ICM group, visual quality scores for DVA1 and DVA2 had significantly higher scores when compared to DSA both overall and in each anatomic region. In the CO_2_ group, DVA values were significantly higher than DSA overall, but only DVA1 was statistically significant in the femoral, popliteal, and crural regions.
Óriás et al., 2020 [17]	26	Carotid percutaneous transluminal angioplasty	Prospective observational study; images were obtained using ND and LD contrast protocols for both DVA and DSA for SNR calculation. Six experts rated image quality of single-image acquisitions on a five-point Likert scale, and compared DVA video fluoroscopy directly with DSA video fluoroscopy through a single questionnaire.	DVA demonstrated a two-fold increase in SNR and outperformed DSA in all single-image comparisons when matched by dose protocol. LD-DVA evaluation scores were statistically unchanged from ND-DSA. Video fluoroscopy comparing low-dose DVA to standard-dose DSA yielded a preference for DVA for diagnostic examination in 61% of comparisons with 69% interrater agreement.
Ansaripour et al., 2024 [18]	n/a	n/a	An economic model based on an average patient population compared costs of DVA versus DSA considering development of acute kidney injury, chronic kidney disease, and radiation-induced cancer over a lifetime.	Analysis concluded that using DVA alongside current imaging practices resulted in savings of an average of 309 English pounds per patient, a QALY gain of 0.025 per patient, and an 8.23% reduction in RR of contrast-associated AKI. Adding DVA to current imaging practices was cost-saving and more effective in 100% of the simulations, even under alternative parameter assumptions.

Clinical Implications

As the use of DVA continues to advance, researchers and clinical practitioners have continued to investigate its diagnostic and clinical implications as compared with DSA. In a retrospective study by Nyárády et al., differences between DSA and DVA were examined among pediatric patients (n = 10, mean age 12) with arteriovenous malformations (AVMs) in the upper and lower limbs, head, neck, and chest [3]. DSA and DVA images were derived from the same angiographic data, but with varying postprocessing techniques. A contrast-to-noise ratio (CNR) was used to assess vessel visibility. Four interventional radiologists and one vascular surgeon (with at least five years of experience) rated image quality on a four-point Likert scale (poor = 1, good = 4) and diagnostic value on a three-point scale (not visible = 1, clear identification = 3). DVA had a significantly higher CNR as compared with DSA but was not superior in clinician visual assessment [3]. For upper limb AVMs, there was no significant difference between techniques. DSA outperformed DVA in imaging of large and small vessels and tissue blush in lower-limb and head/neck AVMs, and in both small and large vessels in chest AVMs. The investigators concluded that DVA is unlikely to provide better diagnostic efficacy as compared with DSA for AVM evaluation. Meanwhile, adjusting DVA's quality reserve may allow less radiation exposure in vulnerable pediatric patients [3].

In a similar study, Lucatelli et al. retrospectively compared DSA and DVA during transarterial chemoembolization (TACE) of liver lesions [4]. Five interventional radiologists with at least 15 years' experience analyzed images in a blinded, randomized review. The goal was to compare the performance of DSA and DVA in liver TACE. Using a four-point Likert scale, DVA demonstrated significantly higher image quality as compared with DSA (p < 0.001) [4]. The researchers also identified significantly fewer instances of non-visible lesions (p < 0.01) and feeding arteries (p < 0.05) with DVA. In paired-image comparisons, 80% of readers preferred DVA over DSA [4].

Bérczi et al. studied the effect of DVA on angiographic quality in transcatheter uterine fibroid embolization (UFE) [5]. DVA was compared with DSA using CNR and visual evaluation by three interventional radiologists (with at least five years of experience) [5]. DVA images showed a two-fold higher median CNR as compared with DSA, which demonstrated significance (p < 0.001) [5]. This increased quality suggests that DVA could reduce radiation dose without sacrificing image quality. Subjective visual comparison using a four-point Likert scale showed no significant clinical difference between DVA and DSA for UFE [5].

Gyánó et al. conducted a prospective study evaluating how CNR affects imaging quality using DVA as compared with DSA, to determine whether DVA’s quality reserve could be used to reduce radiation exposure [6]. Thirty patients with peripheral artery disease were enrolled, and images were obtained using standardized and low-dose contrast protocols for both DVA and DSA in the abdominal, femoral, and crural regions. A total of 472 randomized, blinded images were then provided to seven experts, including three vascular surgeons and four interventional radiologists, who graded the images on a five-point Likert scale [6]. DVA images when statistically analyzed against their same-dose DSA counterpart received higher visual evaluation scores in all anatomical regions (p < 0.05), except for the normal-dose femoral group which had no significant difference [6]. Low-dose DVA images, when statistically compared to normal-dose DSA, had significantly higher CNR ratios in all regions (p < 0.001) [6]. Spearman correlation analysis between normal-dose DSA and low-dose DVA image quality scores demonstrated a strong positive correlation, indicating that low-dose DVA provided superior visualization compared to standard-dose DSA in femoral and crural regions (p < 0.001) [6]. A non-significant difference was observed in the abdominal region because of several outlier data points. Upon re-examination, these outliers were associated with patients who had large amounts of intestinal gas. Since DVA is based on kinetic imaging principles, physiological motion such as bowel movement or gas likely disrupted image quality and accounted for these findings. After excluding these outliers, a significant positive correlation emerged (p = 0.001), indicating that low-dose DVA matched or even exceeded the image quality of standard-dose DSA [6]. Gyánó et al. also highlighted that the overall lowest CNR values were found in the crural region, the same anatomical site as the most statistically significant single-image visual evaluation scores [6]. Visual evaluation of both normal- and low-dose DSA images was the lowest in the crural region, which suggests that DVA may be particularly useful in regions where image quality is generally worse [6].

An adjunctive technology to angiography includes color-coded DSA (ccDSA) and DVA (ccDVA). In this context, color-coding refers to a technique in which colors represent the time and attenuation characteristics of vasculature. Different colors indicate when contrast reaches certain areas of the blood vessels (i.e., red for earlier flow and blue for later flow), while the brightness of a color reflects contrast intensity. This allows clinicians to visualize and measure flow patterns more clearly than traditional gray-scale imaging. To demonstrate the reliability of ccDVA as compared with ccDSA, Góg et al. performed a retrospective comparison of the two modalities using time-related parameters in patients with critical limb-threatening ischemia (CLTI) who underwent lower-limb interventions [7]. The parameters measured included time-to-peak (TTP), which captured the time interval from contrast injection to the moment of maximum contrast intensity within a region of interest, and change in passage time, which provided the difference in the time it takes for the contrast bolus to travel across the target area before and after intervention. Results showed that both time-related parameters in ccDVA were nearly equivalent to those in ccDSA (p < 0.0001) [7].

Alizadeh et al. retrospectively compared DSA with DVA images, and DVA with ccDVA, in the context of prostate artery embolization (PAE) interventions for benign prostatic hyperplasia, using CNR and visual evaluation scores [8]. CNR was calculated for 108 DSA and DVA image pairs, of which DVA images showed a more than four-fold higher CNR value as compared with DSA, which was statistically significant (p < 0.001) [8]. Four interventional radiologists with at least five years of experience evaluated image quality using a four-point Likert scale. This blinded, randomized, single-image evaluation revealed that DVA images had a significantly higher visual score as compared with DSA (p < 0.001) [8]. The readers also compared paired DSA and ccDVA images, which showed a significant preference for ccDVA (p < 0.01), with the best tissue blush appearance [8].

In a case series by Gyánó et al., the researchers compared the diagnostic value of DVA with DSA among four patients who underwent lower-limb angiography with CO_2_ contrast for the treatment of symptomatic peripheral artery disease (PAD) [9]. During each procedure, unsubtracted images were obtained and later utilized to generate both DSA and DVA images. Images within the DSA group underwent postprocessing to achieve optimal image quality for comparison with software-generated DVA images. A retrospective image analysis was performed in a blinded, randomized manner by five evaluators, all with experience in either interventional radiology or vascular surgery. A five-point Likert scale was used to rate images [9]. In single-image review, 84.8% of images were rated significantly higher after DVA processing (p < 0.001), whereas DSA only showed a higher score in only 8.7% of single images [9]. In paired-image review, DVA was the preferred modality over DSA in 85.2% of comparisons for diagnostic value and 83.9% of comparisons for usefulness in therapeutic decision-making [9]. The reviewer’s percent agreement was statistically significant (p < 0.001) [9]. The researchers discussed that DVA’s scoring in image quality was improved even at a frame rate of 1-3 frame(s) per second (FPS), lower than the general recommendation of 4-6 FPS [9].

Óriás et al. also aimed to compare DSA and DVA in the setting of CO_2_ angiography at two separate clinical centers [10]. Researchers prospectively studied 24 patients who underwent lower-limb CO_2_ angiography, using signal-to-noise ratio (SNR) and a visual performance evaluation to compare the two imaging mechanisms [10]. Two SNRs were calculated: SNR of DVA to sumDSA (standard mask subtraction method) and SNR of DVA to maxDSA (maximal intensity projection). SNR was consistently higher in favor of DVA regardless of region, clinical center, or ratio type [10]. For the visual performance evaluation, two vascular surgeons and two interventional radiologists with at least four years of relevant experience compared postprocessed DSA and DVA images as well as the images individually on a five-point Likert scale to assess for image quality [10]. Researchers found that DVA produced a significantly higher image quality than DSA (p < 0.001), regardless of anatomic location [10].

Forty-two individuals were enrolled in a prospective, observational crossover study by Gyánó et al., comparing the SNR image quality and diagnostic capabilities of DVA versus DSA in lower limb angiography [11]. Images were obtained from multiple regions, including the abdomen, iliac, femoral, popliteal, and talocrural areas. Each image set was generated into four image types: raw DSA, postprocessed DSA, raw DVA, and DVA with postprocessing. Three vascular surgeons and three interventional radiologists, each with at least eight years of experience, were blinded and evaluated paired images based on image detail, visibility of anatomical structures, and diagnostic usefulness. When comparing SNR values, the raw DVA demonstrated a 2.3-fold higher SNR across all regions compared to postprocessed DSA, and a 3.3-fold increase compared to raw DSA [11]. In overall image comparison, the evaluators were asked three questions: (1) which image is more detailed, (2) in which image are anatomic structures more discernible, and (3) which image is more useful diagnostically. The answers were statistically analyzed through a percent agreement and Fleiss kappa. The postprocessed DVA was rated superior over summed DSA in 69% of cases, with a statistically significant agreement that DVA was superior in the talocrural and popliteal regions (p < 0.001) and femoral region (p < 0.05) [11]. The researchers concluded that the significant improvement in talocrural and popliteal regions was likely due to the smaller vessel size in these areas where SNR plays a greater role in visibility and diagnosis, as no statistically significant differences were observed in regions with larger vasculature (abdominal and iliac) [11]. In the abdominal region, this was attributed to bowel movements disrupting the motion detection in DVA. In the iliac region, kinetic imaging provided clearer visualization of branching and overlapping vessels, which were more difficult to assess with DSA [11].

In a study by Bastian et al., researchers aimed to evaluate whether DVA could offset artifacts caused by metal implants, thereby improving imaging quality and reducing radiation exposure in patients undergoing lower-limb angiography compared with the current standard of DSA [12]. During angiographic procedures, metal implants can cause radiation scatter, increase image noise, and reduce visualization of adjacent anatomical structures. This scattered radiation contributes to increased radiation exposure for the patient and clinical staff [12]. Twelve patients with various lower-extremity implants were enrolled in the study and underwent lower-limb angiographic procedures for PAD, with the femoral artery as the access point. A minimum of one and up to four image pairs were acquired to evaluate CNR and image quality between DVA and DSA. Three radiologists, with at least four years of relevant experience, evaluated the images independently in a randomized, blinded manner using a paired comparison questionnaire and a five-point Likert scale [12]. The median CNR was two-fold greater in favor of DVA [12]. In the paired comparison of all images (with and without implants), there was a significant preference for DVA in 84.5% of image pairs, with an interrater agreement of 83.2%, and Fleiss kappa value of 0.454 (p < 0.001) [12]. Implant-containing images revealed a preference for DVA in 83.75% of paired image comparisons with a 79.6% interrater agreement and Fleiss kappa value of 0.454 (p < 0.001) [12]. Similarly, all DVA images received significantly higher single-image Likert scores compared to all DSA images (p < 0.001), and the difference in scores for DVA images with implants was significantly higher than their DSA counterpart (p < 0.001) [12].

Dosimetric Implications

Another serious consideration when utilizing angiographic imaging techniques is the potential radiation exposure posed to patients and medical staff. In a prospective, randomized controlled trial, Sótonyi et al. aimed to determine whether the quality reserve of DVA could reduce radiation exposure while maintaining image quality in patients undergoing lower-limb angiography [13]. One hundred and fourteen patients with symptomatic PAD were included and randomly assigned to low-dose (LD) and normal-dose (ND) groups. The ND group was exposed to standard radiation during acquisitions and the LD group's dose and frame value were reduced by 70% [13]. Fluoroscopy settings were kept the same across both groups, and the number of acquisitions did not vary significantly between groups. The ND group's images were processed using DSA, whereas the LD group's images were processed using DSA, DVA1, and DVA2. DVA1 is the original kinetic imaging algorithm. DVA2 is a new algorithm developed to improve image quality when background noise is higher. Blind visual evaluation was performed by six readers (two vascular surgeons and four interventional radiologists with at least seven years of experience) using a five-point Likert scale [13]. The median total dose area product (DAP) and DSA-related DAP reductions in the LD group, when compared to the ND group, were both statistically significant (p < 0.001) [13]. As a whole, LD-DSA images received significantly lower scores from readers as compared with ND-DSA images (p < 0.001). LD-DVA1 images received scores similar to those of ND-DSA images, and LD-DVA2 images received significantly higher scores than ND-DSA images (p < 0.05) [13]. Researchers concluded that DVA can reduce radiation exposure for patients and medical staff during lower-limb angiography procedures without sacrificing image quality or diagnostic value [13].

Secondary analysis of dosimetric exposures from Sótonyi et al. data was completed by Elek et al. in an effort to compare the reduction of radiation between DVA and DSA, and its potential sequelae [13,14]. The collective effective dose for the DSA group was 194.7man x mSv, whereas for the DVA group it was 82.6man x mSv, a reduction of 58% [14]. The data were extrapolated to calculate the lifetime attributable risk (LAR) of cancer incidence and mortality, which was reduced by 25%-75% and 51%-84%, respectively, compared with DSA [14].

Schürmann et al. retrospectively studied the potential reduction in radiation exposure with the use of DVA and the difference in image quality between DVA and DSA among 432 patients [15]. The results demonstrated a significant decrease (p < 0.005) in the median DAP for LD-DSA acquisitions using LD-DVA across all regions [15]. DVA images showed enhanced contrast exposure and reduced background noise across all body regions compared with DSA, even in the LD-DSA group, as objectively quantified by a significantly increased CNR (p < 0.001) [15].

In a study by Thomas et al., researchers retrospectively investigated whether DVA use reduced radiation exposure compared with DSA in the lower limbs [16]. In this study, participants received either iodinated contrast medium (ICM) or CO_2_, and all procedures were performed using standard femoral site access. Two DVA processing algorithms were assessed: DVA1 and DVA2. Unsubtracted serial images were used to generate DSA and DVA datasets, which were then randomized, blinded, and reviewed by six radiologists, who used a five-point Likert scale to grade image quality and diagnostic value [16]. Regardless of the calculation algorithm or contrast medium, DVA images produced significantly higher CNR values as compared with DSA (p < 0.001) [16]. For the ICM group, visual quality scores for DVA1 and DVA2 had significantly higher scores as compared with DSA both overall and in all anatomic regions (p < 0.001) [16]. In the CO_2_ group, DVA values were significantly higher than DSA overall (DVA1, p < 0.001; DVA2, p < 0.05), but only DVA1 was statistically significant in the femoral, popliteal, and crural regions [16]. The researchers concluded that in selective lower limb angiography, regardless of contrast agent, the DVA1 algorithm provided significantly better image quality as compared with DSA [16].

Economic Implications

With a similar aim of assessing whether DVA could provide a safer alternative for patients, Óriás et al. prospectively evaluated whether DVA reduced iodinated contrast medium (ICM) use compared with standard DSA among patients undergoing carotid angiography [17]. Twenty-six patients were enrolled in the study, all of whom underwent carotid percutaneous transluminal angioplasty, with the distal radial artery used as the procedural access site. Standard-dose (100%, 6ml) and low-dose (50%, 3ml) contrast protocols were used to compare DVA and DSA imaging techniques. Image sets were randomized and blinded, then independently assessed by one vascular surgeon and five interventional radiologists using a five-point Likert scale to evaluate image quality [17]. Regardless of the contrast used, DVA demonstrated a two-fold increase in SNR and outperformed DSA in all single-image comparisons when matched by dose protocol (p < 0.001) [17]. Meanwhile, low-dose DVA evaluation scores were statistically unchanged from standard-dose DSA. In addition, video fluoroscopy comparing low-dose DVA to standard-dose DSA yielded a preference for DVA for diagnostic examination in 61% of comparisons with 69% interrater agreement [17]. As such, the researchers suggested that low-dose ICM protocols may be effectively implemented with DVA to potentially reduce the incidence of contrast-related adverse events and associated healthcare costs [17].

An additional parameter discussed by Óriás et al. was the potential economic impact of using DVA to reduce the incidence of contrast-induced nephropathy (CIN) [17]. Researchers hypothesized that the use of imaging modalities associated with fewer renal complications could reduce the incidence of CIN, thereby reducing healthcare costs associated with prolonged hospital stays, morbidity, and mortality [17]. In the United States, an estimated 5%-10% of individuals over the age of 65 years have carotid artery stenosis, and the prevalence is expected to rise [17]. Although DSA remains the standard imaging modality for carotid artery endovascular procedures, researchers suggest that identifying alternative techniques that require lower volumes of ICM, such as DVA, could offer significant economic advantages [17].

The potential economic impact of utilizing DSA versus DVA was studied within the English National Health Service (England, United Kingdom), focusing on the potential avoidance of developing an acute kidney injury (AKI), chronic kidney disease (CKD), and radiation-induced cancer over a given patient's lifetime following the use of DVA [18]. Metrics used to demonstrate potential associated costs include cost per quality-adjusted life-year (QALY), total cost savings per patient, relative risk (RR) reduction in contrast-associated AKI, and lifetime risk of developing carcinoma [18]. The probabilistic base-case analysis concluded that using DVA alongside current imaging practices resulted in savings of an average of 309 English pounds per patient, a QALY gain of 0.025 per patient, and an 8.23% reduction in RR of contrast-associated AKI [18]. In these scenario analyses, the investigators found that adding DVA to current imaging practices was cost-saving and more effective in 100% of the simulations, even under alternative parameter assumptions [18]. Researchers noted that these findings were based on the English healthcare system and may not be applicable to other healthcare systems with vastly different economic and healthcare infrastructures [18].

Discussion

The current literature suggests that DVA is a promising advancement in angiographic imaging. Across diverse clinical contexts, DVA consistently demonstrated non-inferior objective image quality metrics, including CNR and SNR as compared with conventional DSA [4-10]. In many cases, these improvements were associated with greater diagnostic certainty, particularly in scenarios involving smaller or more complex vasculature [6,11].

While most studies included in this review demonstrated that DVA achieves objective image quality measures comparable to, and in some instances exceeding, the current DSA standard, certain works failed to demonstrate a significant advantage in subjective image evaluation. Such studies included the retrospective analysis conducted by Nyárády et al. evaluating AVMs, and the work of Bérczi et al. investigating the use of DVA for UFEs [3,5]. Differences in subjective image evaluation may be due to differences in vascular anatomy, lesion complexity, and the clinical context of each diagnostic task. As subjective assessments are inherently influenced by a myriad of factors including variations in experience and perception, they may not always correlate with objective measures. However, the apparent difference found by certain researchers in subjective compared to objective image quality metrics could have advantageous implications in technically challenging settings including the visualization of complex vasculature and imaging in the presence of metal implants [6,11,12]. Another notable advantage of DVA is its capacity to reduce radiation exposure, while maintaining image quality and diagnostic performance. Three studies included in this review reported significant reductions in DAP, documenting reductions ranging from 38% to 70% [6,13,15]. These reductions could potentially benefit both patients and clinicians during angiographic procedures, particularly for more vulnerable populations including pediatrics and obstetrics. Evidence indicates that DVA image quality can be preserved with lower iodinated contrast volumes, which may be particularly advantageous for patient populations at risk for iodine contrast-associated complications.

Limitations and Future Research

A principal limitation of this review is the relatively small body of literature evaluating DVA used in angiographic procedures. Only 16 studies met the inclusion criteria, and many involved small patient cohorts, potentially limiting the generalizability of their findings. The available evidence was concentrated in a narrow range of clinical applications including lower-limb angiography, TACE of liver lesions, PAE, carotid angiography, and UFE. The performance of DVA across a broader spectrum of angiographic procedures remains insufficiently characterized. Formal biostatistical synthesis was not conducted in this study due to heterogeneity in study designs, endpoints, and outcome measures, which limited the feasibility and interpretability of pooled analysis. This review aimed to provide a structured synthesis of the current evidence rather than definitive conclusions regarding clinical application.

Future research should prioritize large multicenter prospective studies to validate the reported improvements in image quality and reductions in radiation exposure and contrast use. Further investigations into the use of DVA in additional angiographic applications will be necessary to determine whether its benefits extend beyond the currently studied indications and to clarify its potential role in routine clinical practice.

## Conclusions

This review summarizes current data on the use of DVA used during angiographic procedures. DVA has been shown to represent a promising advancement in angiographic imaging, with evidence indicating improved objective image quality metrics and the potential to maintain or enhance diagnostic performance compared to DSA. Across multiple clinical applications, DVA has demonstrated the capacity to significantly reduce radiation exposure and contrast use, suggesting potential improvements in patient safety while maintaining procedural efficacy. Despite these encouraging findings, the current evidence base is limited by small sample sizes and a narrow range of studied applications. Large-scale multicenter studies with standardized protocols and outcome measures will be necessary to validate these results and more clearly define the role of DVA across various vascular territories and procedural settings.
